# Volume control strategy and patient survival in sepsis-associated acute kidney injury receiving continuous renal replacement therapy: a randomized controlled trial with secondary analysis

**DOI:** 10.1038/s41598-024-64224-z

**Published:** 2024-06-21

**Authors:** Cheol Ho Park, Hee Byung Koh, Jin Hyeog Lee, Hui-Yun Jung, Joohyung Ha, Hyung Woo Kim, Jung Tak Park, Seung Hyeok Han, Shin-Wook Kang, Tae-Hyun Yoo

**Affiliations:** 1 Department of Internal Medicine, Institute of Kidney Disease Research, Yonsei University College of Medicine, Seoul, Republic of Korea; 2 Department of Internal Medicine, International Saint Mary’s Hospital, Catholic Kwandong University, Incheon, Republic of Korea

**Keywords:** Bioelectrical impedance analysis, Continuous renal replacement therapy, Sepsis-associated acute kidney injury, Volume control, Nephrology, Renal replacement therapy

## Abstract

Optimal strategy for volume control and the clinical implication of achieved volume control are unknown in patients with sepsis-associated acute kidney injury (AKI) receiving continuous renal replacement therapy (CRRT). This randomized controlled trial aimed to compare the survival according to conventional or bioelectrical impedance analysis (BIA)-guided volume control strategy in patients with sepsis-associated AKI receiving CRRT. We also compared patient survival according to achieved volume accumulation rate ([cumulative fluid balance during 3 days × 100]/fluid overload measured by BIA at enrollment) as a *post-hoc* analysis. We randomly assigned patients to conventional volume control strategy (n = 39) or to BIA-guided volume control strategy (n = 34). There were no differences in 28-day mortality (HR, 1.19; 95% CI, 0.63–2.23) or 90-day mortality (HR, 0.99; 95% CI 0.57–1.75) between conventional and BIA-guided volume control group. In the secondary analysis, achieved volume accumulation rate was significantly associated with patient survival. Compared with the achieved volume accumulation rate of ≤  − 50%, the HRs (95% CIs) for the risk of 90-day mortality were 1.21 (0.29–5.01), 0.55 (0.12–2.48), and 7.18 (1.58–32.51) in that of  − 50–0%, 1–50%, and > 50%, respectively. Hence, BIA-guided volume control in patients with sepsis-associated AKI receiving CRRT did not improve patient outcomes. In the secondary analysis, achieved volume accumulation rate was associated with patient survival.

Acute kidney injury (AKI) is a common and serious complication among critically ill patients in intensive care units (ICUs)^1–3^. The mortality of those critically ill patients with AKI has been reported to reach approximately 60–80%^3–5^. Sepsis is the most common cause of AKI in patients treated in ICUs^6–9^. Patients with sepsis-associated AKI often require continuous renal replacement therapy (CRRT) and have a high risk of mortality^10,11^. Patients with sepsis-associated AKI receiving CRRT often experience fluid overload and recent studies have shown a relationship between fluid overload or cumulative fluid balance and adverse outcomes in critically ill patients^12–15^.

Owing to the poor prognosis of patients with sepsis-associated AKI requiring CRRT, a number of studies have been conducted to find an optimal strategy for prescribing CRRT, including the timing of the initiation and dosing of kidney support^16–23^. However, clinical trials designed to evaluate the optimal approach to guide fluid removal using CRRT in these patients are still lacking. Additionally, information on the association between the resolution of fluid overload and clinical outcomes is relatively scarce.

Accurate volume status evaluation is essential for the appropriate management of patients with excessive body fluid receiving CRRT. Conventionally, a review of daily fluid balance, physical examination, and chest radiography are used to estimate the amount of excess fluid in critically ill patients with AKI. However, these approaches are considered inaccurate^24–26^. Bioelectrical impedance analysis (BIA) is a simple and relatively reliable method for estimating the body composition, particularly for detecting fluid overload^27–29^. However, the effect of BIA on the management of patients with sepsis-associated AKI receiving CRRT and accompanying fluid overload remains unclear.

By conducting a prospective randomized controlled trial with secondary analysis, this study examined whether BIA-guided volume control using CRRT could improve patient survival and the association between the achieved volume accumulation and clinical outcomes in patients with sepsis-associated AKI.

## Results

### Baseline characteristics according to volume control strategies

The baseline characteristics of the 73 participants according to volume control strategy are presented in Table 1. The baseline characteristics were similar between the two groups. The median age was 67 (IQR, 58–74) years, and 53.4% of the patients were male. Approximately one-third (31.5%) had diabetes mellitus. Respiratory infection attributed 47.9% for the cause of sepsis cases. The median APACHE II and SOFA scores were 32 (IQR, 26–35) and 12 (IQR, 11–16), respectively. Overall, 79.5% of the patients required mechanical ventilation support. Median estimated glomerular filtration rate (eGFR) before AKI onset was 79 (IQR, 40–97) mL/min/1.73 m^2^. The volume overload assessed using BIA was similar between the groups.

**Table 1:** Baseline characteristics and the risk of outcomes according to volume control strategies.

	Total N = 73	Conventional volume control N = 39	BIA-guided volume control N = 34	*P* value
Baseline characteristics				
Age, yr	67 [58–74]	68 [58–75]	67 [56–73]	0.83
Male, n (%)	39 (53.4)	21 (53.8)	18 (52.9)	0.94
BMI, kg/m^2^	23.9 (3.8)	23.9 (3.9)	23.9 (3.8)	0.95
Comorbid diseases, n (%)				
Hypertension	34 (46.6)	19 (48.7)	15 (44.1)	0.69
Diabetes mellitus	23 (31.5)	11 (28.2)	12 (35.3)	0.52
Ischemic heart disease	8 (11.0)	6 (15.4)	2 (5.9)	0.19
Chronic kidney disease	37 (50.7)	20 (51.3)	17 (50.0)	0.91
Type of infection, n (%)				0.57
Respiratory	35 (47.9)	20 (51.3)	15 (44.1)	
Gastrointestinal	16 (21.9)	8 (20.5)	8 (23.5)	
Genitourinary	4 (5.5)	1 (2.6)	3 (8.8)	
Musculoskeletal	1 (1.4)	0 (0.0)	1 (2.9)	
Others	17 (23.3)	10 (25.6)	7 (20.6)	
Mechanical ventilation, n (%)	58 (79.5)	30 (76.9)	28 (82.4)	0.57
Vasoactive-inotropic score	41.6 [17.1–69.5]	39.0 [11.9–69.5]	41.9 [18.5–70.0]	0.55
Vasopressor dependency index, mmHg^−1^	0.5 [0.2–0.9]	0.5 [0.2–1.2]	0.5 [0.2–0.9]	0.62
APACHE II score	32 [26–35]	32 [26–36]	31 [24–34]	0.13
SOFA score	12 [11–16]	12 [10–15]	13 [11–16]	0.49
eGFR, mL/min/1.73m^2^				
Preadmission	79 [40–97]	81 [36–105]	72 [47–91]	0.83
At CRRT initiation	22 [15–36]	23 [13–38]	21 [16–33]	0.99
Mean arterial pressure, mmHg	76 (13)	76 (14)	76 (13)	0.83
Urine output for 2 h before CRRT initiation, mL	20 [5–60]	13 [0–55]	25 [10–65]	0.12
White blood cells, × 1000 cells/μL	14.07 (11.00)	14.12 (8.80)	14.03 (13.23)	0.97
Hemoglobin, g/dL	9.2 (2.0)	9.4 (2.0)	8.9 (2.0)	0.29
Blood urea nitrogen, mg/dL	66.1 (31.4)	60.3 (25.5)	72.8 (36.3)	0.091
Albumin, g/dL	2.5 (0.4)	2.6 (0.5)	2.5 (0.3)	0.25
C-reactive protein, mg/dL	116.6 (105.1)	112.4 (115.8)	121.4 (93.3)	0.73
pH	7.27 (0.14)	7.26 (0.13)	7.28 (0.14)	0.53
Lactate, mmol/L	5.1 (4.5)	5.1 (4.9)	5.0 (4.0)	0.91
Volume overload, L	3.7 [2.3–7.0]	3.7 [2.4–6.6]	4.2 [1.8–8.2]	0.41
CRRT duration, days	5.0 [3.0–10.0]	6.0 [3.0–12.0]	5.0 [2.0–8.0]	0.47
Outcomes				
28-day mortality, n (%)	39 (53.4)	20 (51.3)	19 (55.9)	0.69
90-day mortality, n (%)	49 (67.1)	27 (69.2)	22 (64.7)	0.68
ICU death, n (%)	42 (57.5)	20 (51.3)	21 (61.8)	0.37
In-hospital death, n (%)	53 (72.6)	31 (79.5)	22 (64.7)	0.16
7-day mortality, n (%)	20 (27.4)	10 (25.6)	10 (29.4)	0.69
Hazard ratios (95% confidence intervals)				
28-day mortality		1.00	1.19 (0.63–2.23)	0.591
90-day mortality		1.00	0.99 (0.57–1.75)	0.984
ICU death		1.00	1.20 (0.65–2.22)	0.557
In-hospital death		1.00	0.99 (0.57–1.73)	0.978
7-day mortality		1.00	1.19 (0.50–2.86)	0.697

Data are expressed as mean (standard deviation), median [interquartile range], or count (%).

*APACHE* Acute physiology and chronic health evaluation; *BIA* bioelectrical impedance analysis; *BMI* body mass index; *CRRT* continuous renal replacement therapy; *eGFR* estimated glomerular filtration rate; *ICU* intensive care unit; *SOFA* Sequential oran failure assessment.

### Volume reduction, fluid balance and CRRT prescription status according to volume control strategies

Table 2 shows volume reduction, fluid balance, and CRRT prescription status. The BIA-guided volume control strategy achieved target daily volume reduction in 21.7–26.5% of subjects, whereas the conventional volume control strategy reduced one-third of overhydration in 9.4–13.5% of subjects daily. Over 3 days, patients allocated to the BIA-guided volume control strategy appeared to have a lower fluid balance than those allocated to the conventional volume control strategy. In contrast, the ultrafiltration volumes during the intervention period were 2,222 (SD, 796) mL/day with the conventional volume control strategy and 2,723 (SD, 1,114) mL/day with the BIA-guided volume control strategy (*P*-value 0.03). Additionally, the ultrafiltration rates were 36.2 (SD: 13.7) mL/kg/day and 44.6 (SD: 19.5) mL/kg/day, respectively (*P*-value 0.04). The mean CRRT doses were 36.9 (SD, 3.7) mL/kg/h in the conventional volume control group and 38.5 (SD, 3.1) mL/kg/h in the BIA-guided volume control group, showing a marginal statistical difference (*P*-value 0.06).

**Table 2:** Volume control, fluid balance, and CRRT prescription status in the two treatment groups during the intervention.

	Total N = 73	Conventional volume control N = 39	BIA-guided volume control N = 34	*P* value
Target volume achievement, n (%)				
Day 1	13 (17.8)	4 (10.3)	9 (26.5)	0.07
Day 2	13 (19.1)	5 (13.5)	8 (25.8)	0.20
Day 3	8 (14.5)	3 (9.4)	5 (21.7)	0.20
Fluid balance, mL				
Day 1	498 [-403–1906]	1075 [-403–1986]	179 [-542–1217]	0.15
Day 2	122 [-420–1028]	219 [-366–1080]	79 [-757–843]	0.57
Day 3	13 [-586–681]	34 [-510–560]	-9 [-835–799]	0.54
Day 1–3	797 [-760–2840]	1302 [-391–2866]	586 [-1350–2840]	0.19
CRRT ultrafiltration volume, mL				
Day 1	2363 (1218)	1969 (945)	2816 (1347)	0.002
Day 2	2609 (1225)	2538 (1175)	2693 (1296)	0.61
Day 3	2416 (1177)	2187 (976)	2736 (1370)	0.09
Day 1–3	6614 (3198)	6171 (2551)	7123 (3783)	0.21
CRRT status				
CRRT ultrafiltration volume, mL/day	2455 (983)	2222 (796)	2723 (1114)	0.03
CRRT ultrafiltration rate, mL/kg/day	40.1 (17.0)	36.2 (13.7)	44.6 (19.5)	0.04
CRRT dose, mL/kg/h	37.6 (3.5)	36.9 (3.7)	38.5 (3.1)	0.06

Data are expressed as mean (standard deviation), median [interquartile range], or count (%).

*BIA* bioelectrical impedance analysis; *CRRT* continuous renal replacement therapy.

### Patient outcomes according to volume control strategies

**Figure 1:**
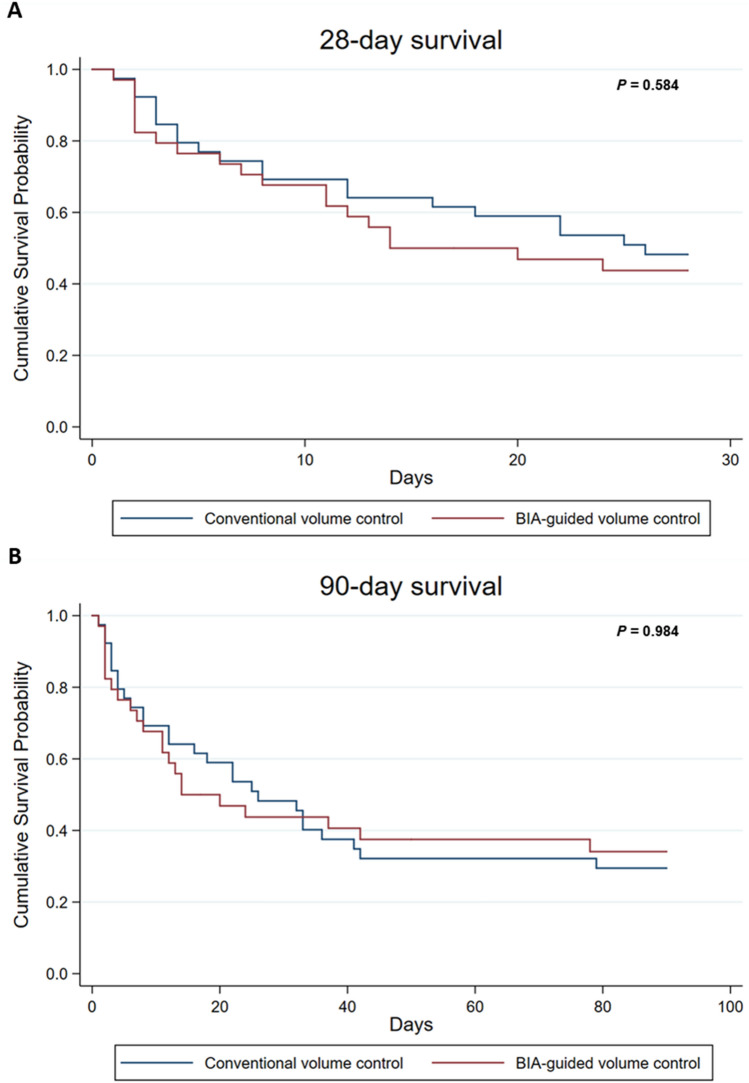
Kaplan–Meier curves showing patient survival according to volume control strategies. Cumulative survival probability within (**A**) 28-day and (**B**) 90-day of continuous renal replacement therapy initiation according to volume control strategies. Log-rank tests were used for comparison between groups. *BIA* bioelectrical impedance analysis.

Overall, 20 of the 39 (51.3%) patients in the conventional volume control group died within 28 days of randomization compared to 19 of the 34 patients (55.9%) in the BIA-guided volume control group. By day 90, 27 (69.2%) patients had died in the conventional volume control group compared with 22 (64.7%) in the BIA-guided volume control group (Table 1). Survival rates at 28 (*P* = 0.584) and 90 (*P* = 0.984) days after randomization did not differ significantly between the treatment groups (Fig. 1). Additionally, there was no difference in the rates of ICU and in-hospital deaths and 7-day mortality between the treatment groups (Table 1 and Supplementary Fig. S1).

In a univariate Cox regression analysis, BIA-guided volume control dsid not significantly improve 28-day (HR, 1.19; 95% CI, 0.63–2.23; *P* = 0.591) nor 90-day (HR, 0.99; 95% CI 0.57–1.75; *P* = 0.984) survival. In like manner, the BIA-guided volume control group did not show a difference in ICU death (HR, 1.20; 95% CI 0.62–2.22; *P* = 0.557), in-hospital death (HR, 0.99; 95% CI 0.57–1.73; *P* = 0.978), nor 7-day survival (HR, 1.19; 95% CI 0.50–2.86; *P* = 0.697) (Table 1).

Additionally, we compared the VIS and VDI statuses according to volume control strategies. The VIS and VDI scores did not significantly differ between the volume control strategies during the randomization period. In addition, the proportion of participants who experienced an increase (≥ 30% or ≥ 50%) in the VIS from baseline was similar between the two treatment arms (Supplementary Table  S1).

### Trajectories of clinical parameters according to volume control strategies

Clinical parameters that may be affected by volume control strategies are presented in Table 3. There were no differences in the average systolic blood pressure, the occurrence of hypotension, urine output, pH, lactate level, and total CO_2_ level between study groups.

**Table 3:** Clinical parameters tin the two treatment groups during the intervention.

	Total N = 73	Conventional volume control N = 39	BIA-guided volume control N = 34	*P* value
Systolic blood pressure, mmHg				
Day 1	121 (22)	120 (22)	122 (23)	0.81
Day 2	117 (24)	117 (22)	118 (27)	0.95
Day 3	121 (27)	125 (29)	117 (25)	0.26
Hypotension, n(%)				
Day 1	45 (61.6)	26 (66.7)	19 (55.9)	0.34
Day 2	31 (45.6)	16 (43.2)	15 (48.4)	0.67
Day 3	30 (51.7)	15 (48.4)	15 (55.6)	0.59
Urine output, mL/day				
Day 1	185 [51–585]	140 [35–605]	225 [88–485]	0.39
Day 2	78 [15–190]	75 [15–180]	90 [45–190]	0.65
Day 3	45 [13–210]	25 [10–210]	113 [26–213]	0.14
pH				
Day 1	7.41 [7.35–7.45]	7.41 [7.35–7.46]	7.41 [7.35–7.45]	0.84
Day 2	7.42 [7.35–7.45]	7.41 [7.33–7.46]	7.42 [7.35–7.45]	0.81
Day 3	7.42 [7.37–7.49]	7.44 [7.36–7.50]	7.41 [7.37–7.47]	0.56
Lactate, mmol/L				
Day 1	2.7 [1.9–9.2]	3.1 [1.9–10.9]	2.4 [1.8–8.2]	0.36
Day 2	2.3 [1.5–7.0]	1.9 [1.6–7.9]	2.5 [1.4–5.0]	0.66
Day 3	2.0 [1.4–4.8]	4.3 [1.4–5.3]	1.9 [1.4–2.9]	0.46
Total CO_2_				
Day 1	22.7 [20.8–25.2]	22.4 [21.5–25.8]	22.9 [20.2–24.7]	0.54
Day 2	23.1 [21.8–25.9]	23.5 [22.1–26.3]	22.9 [21.6–25.2]	0.45
Day 3	23.3 [21.5–24.9]	23.9 [21.9–25.1]	22.7 [21.5–24.1]	0.26

Data are expressed as mean (standard deviation), median [interquartile range], or count (%).

*BIA* bioelectrical impedance analysis.

### Baseline characteristics according to achieved volume accumulation rate categories

The baseline characteristics of the 55 participants according to volume accumulation rate categories are presented in Supplementary Table S2. The overall demographic characteristics were similar to those in the primary analysis. Patients with high volume accumulation rates had higher VIS and VDI values, indicating hemodynamic instability. There were no differences in the APACHE II scores, SOFA scores, and various laboratory parameters.

### Patient outcomes

A total of 26 (47.2%) and 35 (63.6%) deaths occurred within 28 days and 90 days of CRRT initiation, respectively (Supplementary Table S3). ICU death and in-hospital death occurred in 27 (49.1%) and 39 (70.9%) patients, respectively. Patients with the highest achieved volume accumulation rate (> 50%) showed significantly higher rate of 28-day mortality (*P* < 0.001) and 90-day mortality (*P* < 0.001). Additionally, the rates of ICU death (*P* < 0.001) and in-hospital death (*P* < 0.001) were significantly higher in the group with the highest achieved volume accumulation rate (> 50%).

### Association of achieved volume accumulation rate with the patient survival

Kaplan–Meier curves revealed that the cumulative 28- and 90-day survival probabilities were significantly lower for patients in the achieved volume accumulation range of > 50% than for the others (*P* < 0.001) (Supplementary Fig. S2).

The association between the achieved volume accumulation rate and patient survival was further evaluated using multivariate Cox proportional hazard models. In the unadjusted model, the HRs (95% CIs) for the risk of 28- and 90-day mortality in the participants with the achieved volume accumulation rate of > 50% were 4.96 (1.60–15.38) and 5.62 (2.01–15.66), respectively, compared to the participants with the achieved volume accumulation rate of ≤ -− 50% (model 1 in Supplementary Table S4). The association between the achieved volume accumulation rate and patient survival was maintained after adjusting for demographic factors, infection type, intervention arm, VDI, APACHE II score, fluid overload, and serum lactate levels (Models 2 and 3 in Supplementary Table S4). Further adjustment of the CRRT prescription status did not change the increased risks of 28- and 90-day mortality in patients with the highest achieved volume accumulation rates (> 50%). The corresponding HRs (95% CIs) for subjects with the achieved volume accumulation rate of > 50% were 8.08 (1.18–55.50) (28-day mortality) and 7.18 (1.58–32.51) (90-day mortality) (model 4 in Supplementary Table S4). In continuous modeling, a 10-% increase in achieved volume accumulation rate was associated with marginally increased risk for 28-day mortality (HR, 1.06; 95% CI 0.99–1.12; *P* = 0.076) and significantly increased risk for 90-day mortality (HR, 1.05; 95% CI 1.00–1.05; *P* = 0.048) (model 4 in Supplementary Table S4).

We also confirmed the association between the achieved volume accumulation rate and patient survival using ICU and in-hospital deaths as outcomes. In the fully adjusted model, the HRs (95% CIs) for the risk of ICU death and in-hospital death for the patients with the achieved volume accumulation rate of > 50% were 19.33 (2.99–125.08) and 8.99 (2.05–39.45), respectively, compared to patients with the achieved volume accumulation rate of ≤  − 50% (model 4 in Supplementary Table S4). In continuous modeling, a 10-% increase in achieved volume accumulation rate was associated with a 1.09-fold (95% CI, 1.02–1.16) higher risk of ICU death and 1.06-fold (95% CI, 1.01–1.11) increased irk of in-hospital death (model 4 in Supplementary Table S4).

## Discussion

In the present study, the BIA-guided volume control strategy in patients with sepsis-associated AKI receiving CRRT did not result in a significantly higher rate of target volume reduction or improved clinical outcomes, including survival, compared to conventional volume control strategies. In contrast, the achieved volume accumulation rate, as assessed using BIA and accumulative fluid balance, was associated with patient survival.

Recent studies have shown that mortality rates are > 50% in critically ill patients with AKI, and these poor outcomes are usually attributed to multiple complications associated with AKI, including infection, bleeding, electrolyte imbalance, and fluid overload^30,31^. CRRT is an established treatment modality for patients with AKI in intensive care units. Many investigations have compared CRRT prescription strategies regarding the timing of initiation and dose of kidney support to improve clinical outcomes^16–22^. However, information on the prescription strategies of CRRT for fluid overload derived from randomized studies is relatively scarce despite the significance of fluid accumulation in adverse outcomes of patients with AKI or critical illness^12,32,33^. Bouchard et al.^12^ revealed that fluid overload was independently associated with survival in patients with AKI in the Program to Improve Care in Acute Renal Disease (PICARD). Recently, a meta-analysis involving 31,076 from 31 observational and three randomized controlled trials highlighted that fluid overload and positive cumulative fluid balance were associated with increased mortality in critically ill patients^33^. Furthermore, a decrease in cumulative fluid balance was associated with a lower risk of mortality in a cohort study of patients receiving CRRT reported by Hall et al.^34^ On the other hand, information supported by clinical trials is limited. The Initiation of Dialysis Early Versus Delayed in the Intensive Care Unit (IDEAL-ICU) resulted in a nominally high prevalence of fluid overload and greater cumulative fluid balance in the delayed strategy but did not reach statistical significance^18^. Additionally, a post hoc analysis of Standard versus Accelerated Initiation of Renal-Replacement Therapy in AKI (STARRT-AKI) study showed that the accelerated strategy for CRRT initiation modestly reduced the cumulative fluid balance during the two weeks following randomization^35^. However, these studies did not focus on volume control strategies in critically ill patients undergoing CRRT.

In this study, we conducted a randomized controlled trial primarily aimed at examining the volume control strategy in patients undergoing CRRT (BIA-guided volume control *versus* conventional volume control). Although the BIA-guided volume control strategy conferred a greater nominal ultrafiltration volume and rate than the conventional volume control strategy throughout the intervention period, the BIA-guided volume control did not demonstrate a significantly higher proportion of target volume reduction and failed to improve patient outcomes in this study. One explanation for these results could be that the maintenance of organ perfusion through the achievement of hemodynamic stability is more crucial than the resolution of fluid overload for improving survival in patients with sepsis-associated AKI receiving CRRT. Another possibility is that the removal of excessive fluid using the BIA-guided method was insufficient to result in favorable clinical outcomes compared to the conventional method, even though the former yielded a greater ultrafiltration rate than the latter. Additionally, the clinical outcomes of patients might be more dependent on the progress of infection than on the volume status of the patients. Lastly, insufficient study size might be a reason for negative results.

In the secondary analysis, the achieved volume accumulation rate was associated with patient survival. In particular, an achieved volume accumulation rate of > 50% indicated that a cumulative fluid balance during 3 days over 50% of fluid overload at CRRT initiation was associated with poor prognosis. In line with our findings, in an Intensive Care Over Nations (ICON) study involving over 1800 patients admitted to the ICU with sepsis from 84 countries, a higher cumulative fluid balance on day 3 was independently associated with an increased risk of mortality^14^. In addition, the Role of Active Deresuscitation After Resuscitation (RADAR) investigators showed that fluid balance was associated with a higher risk of 30-day mortality and that achieving negative fluid balance on day 3 of ICU admission was associated with improved patient survival in patients receiving invasive mechanical ventilation^15^. However, these studies did not consider the relationship between the cumulative fluid balance and the amount of fluid overload at baseline. Our study results verified the association between cumulative fluid balance and survival in patients with sepsis-associated AKI receiving CRRT using a novel metric that reflects cumulative fluid balance and the patient’s initial status of fluid overload.

Incorporating the results from intention-to-treat analysis and secondary analysis, our study suggests some clinical implications of using BIA in managing patients with AKI undergoing CRRT. Considering that BIA-guided volume control resulted in nominally larger ultrafiltration volume compared to conventional volume control without prompting hemodynamic instability or hindering tissue perfusion, BIA may provide further information for appropriate volume control in patients with AKI undergoing CRRT when compared to volume control solely depending on clinical parameters. Furthermore, the implementation of BIA in the management of those patients could confer additional prognostic information.

This study had several limitations. First, this study may have been underpowered for clinical outcomes owing to the relatively small number of participants. This could be attributed to the high dropout rate for various reasons. The major reasons for dropout were withdrawal of consent for the study and withdrawal by the attending physician. The retractions by patients (or substitute decision-makers) may be due to cultural background or deterioration of patients’ medical condition. The withdrawals from the study by the attending doctors were mainly attributed to the disagreement regarding the volume control strategy suggested by consulting nephrologists. Second, most participants in the intervention arm (BIA-guided volume control strategy) did not reach the target volume reduction, and the proportion of participants who accomplished the target was not significantly higher in the BIA-guided volume control group than in the conventional volume control group. This could be explained by the hemodynamic instability of the participants, who required a high dose of vasoactive drugs. Thus, the implementation of intensive volume control might have been difficult for participants. Although the proportions of target volume reduction achievement were not significantly different, the BIA-guided volume control group showed an approximately two-fold higher nominal rate of target volume reduction. Therefore, our findings provide insights into volume control strategies in patients with sepsis-associated AKI requiring CRRT. Third, our study was conducted at a single center. Fourth, the open-label design may have introduced bias in patient management. Fifth, although the consulting nephrologists’ decisions for CRRT initiation were not significantly different, the exact criteria or timing of CRRT initiation may have varied among the participants. However, considering that clinical parameters affect the decision for initiating CRRT, such as urine output, laboratory values including eGFR, and disease severity indices, were comparable between the study groups, timing of CRRT initiation did not differ between the volume control strategy. Sixth, residual confounding factors may have been present in the secondary analysis. To overcome this issue, we adjusted for confounding factors that might have affected the outcomes. Finally, our study enrolled only Korean patients. Thus, our study results cannot be extrapolated to patients with ethnic backgrounds other than Korean.

In conclusion, the BIA-guided volume control strategy did not yield greater volume removal or improved patient outcomes compared with the conventional volume control strategy in patients with sepsis-associated AKI requiring CRRT. However, our study revealed that the achieved volume accumulation rate was associated with patient survival. The volume accumulation rate achieved may be a useful metric for the management of patients with sepsis-associated AKI receiving CRRT.

## Methods

### Study setting

We conducted a prospective, single-center, open-label, randomized controlled trial that assessed BIA-and clinical criteria-guided volume control using CRRT in patients with sepsis-associated AKI in the ICU of a large tertiary care hospital (Severance Hospital, Seoul, Republic of Korea) (Trial registration: ClinicalTrials.gov, NCT02384525, Registered 10 March 2015, https://clinicaltrials.gov/study/NCT02384525). This study was conducted between June 2017 and October 2021. This study was approved by the institutional review board of Severance Hospital and conducted in accordance with the provisions of the Declaration of Helsinki (institutional review board approval number:4-2014-0791). All participants and/or substitute decision-makers were informed of the study and provided written informed consent.

### Participants selection

Participants were eligible for enrollment if they were aged 19 years or older, admitted to the ICU with AKI due to sepsis, and required CRRT. Each case of sepsis was defined according to the consensus conference criteria suggested by the Society of Critical Care Medicine and American College of Chest Physicians^36^. Briefly, if a patient had a suspected infection and met systemic inflammatory response syndrome criteria (2 or more of following; body temperature < 36 °C or > 38 °C, heart rate > 90 beats/min, respiratory rate > 20 breaths/min or PaCO2 < 32 mmHg, or white blood cell count < 4.0 × 10^3^/μL or > 12.0 × 10^3^/μL) in two consecutive measurements, we diagnosed sepsis. Infection was diagnosed if the causative organisms were isolated by culture studies or were clinically suspected as follows: (1) white blood cells in a normally sterile fluid; (2) perforated viscus; or (3) obvious evidence of infection from imaging tests, including pneumonia and abscess. We included patients with AKI at a level greater than the ‘injury’ stage according to the RIFLE criteria, which was consistent with a more than twofold increase in serum creatinine level compared with baseline or urine output < 0.5 mL/kg/h over 12 h. The exclusion criteria were as follows: (1) patients older than 80 years; (2) patients already receiving kidney replacement therapy due to kidney failure; (3) life expectancy of less than 3 months due to terminal cancer; (4) presence of an intracardiac device, including a pacemaker, implantable cardioverter defibrillator, or cardiac resynchronization therapy; (5) pregnancy or lactation; or (6) generalized exfoliative skin disease. Participants were excluded from the final analysis if their severe hypophosphatemia (serum phosphorus < 2.5 mg/dL) or hypokalemia (serum potassium < 3.5 mg/dL) was not corrected within 12 h after the first detection.

### Data collection and measurements

The baseline demographic, clinical, and biochemical characteristics were collected at the time of randomization. Disease severity was determined using the Acute Physiology and Chronic Health Evaluation (APACHE) II and Sequential Organ Failure Assessment (SOFA) scores. We assessed vital signs, doses of vasoactive/vasopressor agents, and laboratory test results, including complete blood count, chemistry, blood gas analyses, and lactic acid levels. The dose of inotropic agents was expressed as the vasoactive-inotropic score (VIS) and vasopressor dependency index (VDI), which is a dimensionless variable calculated using the following formulas^37–39^.

$$\begin{aligned} VIS & = dopa\min \;dose \left( {\mu g/kg/\min } \right) + dobuta\min e \;dose \left( {\mu g/kg/\min } \right) + 100; & \\ & \quad \times epinephrine\; dose \left( {\mu g/kg/\min } \right) + 10 \times \min irone\; dose \left( {\mu g/kg/\min } \right) \\ & \quad + 10000 \times vasopres\sin \; dose \left( {units/kg/\min } \right) + 100 \\ & \quad \times norepinephrine\; dose \left( {\mu g/kg/\min } \right) \\ \end{aligned}$$

$$VDI = \frac{VIS}{{Mean\;arterial\;pressure \left( {mmHg} \right)}}$$

Serum creatinine levels were measured using an isotope-dilution mass spectrometry-tractable method, and the estimated glomerular filtration rate (eGFR) was calculated using the Chronic Kidney Disease Epidemiology Collaboration equation^40^.

### BIA measurement

The amount of fluid overload was assessed by BIA using a Body Composition Monitor (BCM) (Fresenius Medical Care, Bad Homburg vor der Hohe, Germany) according to the manufacturer’s instructions within 6 h of CRRT initiation. Briefly, the participants were removed from the metallic devices or accessories. The BCM electrodes were then placed on the dorsum of the hand and foot at the metacarpal and metatarsal sites, respectively. The parameter overhydration was adopted for the amount of fluid overload.

### Treatment assignments

CRRT was initiated at the discretion of a consulting nephrologist without considering the patient’s eligibility for this study. Generally, CRRT is prescribed in patients with AKI at a stage greater than the injury stage classified by the RIFLE criteria, with the presence of significant volume overload, uncontrolled hyperkalemia (potassium > 6.5 mEq/L), or severe acidemia (pH < 7.2). CRRT was delivered by Prisma or Prisma Flex machines (Baxter, Deerfield, IL, USA) using ST100 (surface area, 1.0 m^2^) filter sets. Vascular access for CRRT was obtained by inserting a 14F double-lumen catheter into the internal jugular or femoral vein. The effluent volume was set to achieve clearance rate of 40 mL/kg/h for guaranteeing minimal clearance rate of 35 mL/kg/h considering interruption of CRRT due to special examinations (e.g. computed tomography) or unexpected causes (e.g. machine error)^22^. All patients received CRRT in the continuous veno-venous hemodiafiltration mode. The replacement and dialysate volumes were set using a 1:1 balanced predilution method^22^. In the conventional volume control group, volume control was performed by an experienced nephrologist based on clinical parameters such as daily fluid balance, physical examination, and review of chest radiographs. In the BIA-guided volume control group, the amount of daily volume reduction was determined to be one-third of overhydration, and the strategy of volume control was maintained for 72 h (Supplementary Fig. S3). After 72 h of randomization, volume control in both groups was performed at the discretion of an experienced nephrologist.

Patients remained on CRRT until kidney function recovered, transfer to conventional hemodialysis, withdrawal of CRRT as part of life support, or death. The decision to wean a patient from CRRT was made by a nephrologist when the patient recovered hemodynamic stability during intermittent hemodialysis or had considerable urine output.

Patients eligible for enrollment were informed of the study and those who provided written consent were randomly assigned in a 1:1 ratio to each treatment group using a centralized computer-generated adaptive randomization scheme by a neutral person who was not involved in the trial at the time of CRRT initiation.

### Sample size estimation and study population

**Figure 2:**
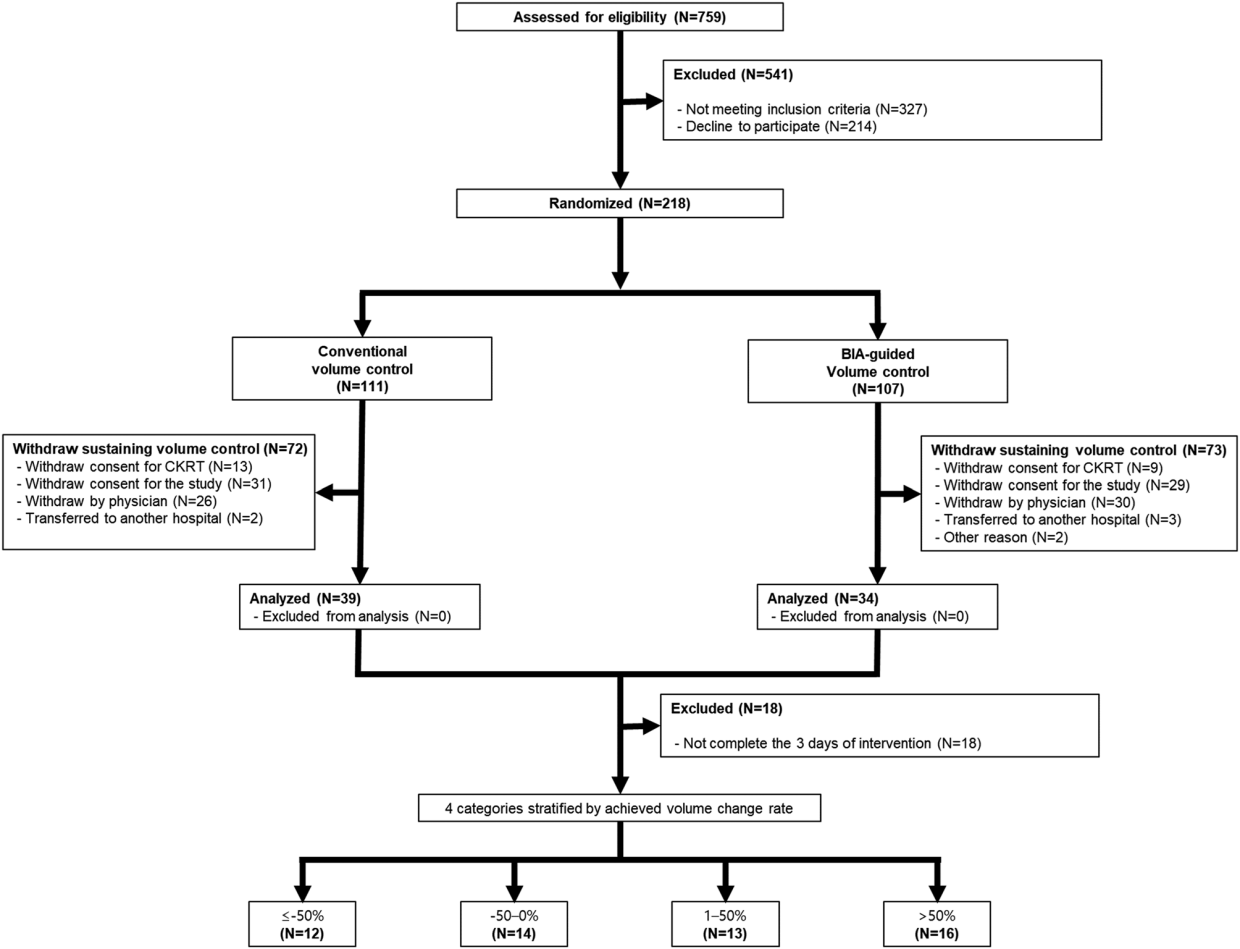
Flow diagram of study participants. From June 2017 to October 2021, a total of 759 patients who initiated continuous renal replacement therapy at Severance Hospital (Seoul, Republic of Korea) were initially assessed for eligibility. According to inclusion and exclusion criteria, 73 patients were included in the primary analysis (intention-to-treat analysis). After excluding 18 patients who did not complete the 3 days of intervention, 55 subjects were included in the secondary analysis. *BIA* bioelectrical impedance analysis; *CRRT* continuous renal replacement therapy.

The sample size was estimated based on the following assumptions. The mortality rate in the control group was estimated to be 60% based on previous studies^22,41,42^. This study aimed to demonstrate a ≥ 20% reduction in mortality rate. To detect a significant difference in the primary outcome with a two-sided type I error of 0.05 and 80% power, we estimated that 82 participants would be required for each arm. We aimed to enroll at least 109 patients to allow a dropout rate of 25% in individual treatment groups. A total of 73 patients were included in the primary analysis (intention-to-treat analysis) (Fig. 2). In the secondary analysis to investigate the association between the achieved volume accumulation rate and clinical outcomes, we excluded 18 patients who did not complete the 3 days of intervention. Finally, 55 participants were included in the secondary analysis (Fig. 2).

### End points

The outcome of interest was death from any cause within 28 and 90 days of randomization. ICU and in-hospital deaths were also evaluated. ICU death and in-hospital death were defined as death during the ICU stay or hospitalization, regardless of the time point. Additionally, we set death from any cause within 7 days of randomization as a short-term outcome.

### Statistical analysis

The data were analyzed using Stata 15.1 (Stata Corporation). All data are expressed as the mean (standard deviation [SD]) or median (interquartile range [IQR]). The *t* test of the Mann–Whitney *U* test was used for continuous variables, and the chi-square test was used for categorical variables. Univariate Cox proportional hazards analysis was conducted to compare survival between the conventional and BIA-guided volume control groups at 28 and 90 days after randomization, as well as the aforementioned secondary endpoints. In the secondary analysis, we compared the risk of patient survival according to the achieved volume accumulation rate over 3 days. The volume accumulation rate was calculated using the following equation:

$$Achieved\,volume\, accumulation\, rate = \frac{Cumulative\,fluid \,balance \,for \,3 \,days \,after\, randomization}{{Fluid \,overload \,measured \,by\, BIA \,at \,enrollment}} \times 100$$

The achieved volume accumulation rate was analyzed as follows: (1) a categorical variable in which the achieved volume accumulation rate was stratified into 4 groups (≤  − 50%,  − 50–0%, 1–50%, and > 50%) and (2) a continuous variable in 10-% increments.

The Cox proportional hazards model was used for the analysis. We made incremental adjustments with the following variables: Model 1 is an unadjusted model. Model 2 was adjusted for age, sex, body mass index, type of infection, and intervention arm. We added the VDI, APACHE II score, fluid overload, and serum lactate level to Model 3. In Model 4, the CRRT ultrafiltration rate and CRRT dose were added. The results of Cox proportional hazards regression are presented as hazard ratios (HRs) and 95% confidence intervals (CIs).

Data analyses were performed by an independent statistician blinded to the treatment assignment. *P* < 0.05 was considered statistically significant.

### Ethics approval

This study was approved by the institutional review board of Severance Hospital with registration number 4-2014-0791, and was registered at http://www.clinicaltrials.gov with the trial registration number NCT02384525.

### Informed consent

Written informed consent was obtained from all participants and/or substitute decision makers.

### Supplementary Information

Supplementary Information.

## Data Availability

The datasets generated during and/or analyzed in the current study are available from the corresponding author upon reasonable request.
