# Author Correction: Volume control strategy and patient survival in sepsis-associated acute kidney injury receiving continuous renal replacement therapy: a randomized controlled trial with secondary analysis

**DOI:** 10.1038/s41598-024-70484-6

**Published:** 2024-08-22

**Authors:** Cheol Ho Park, Hee Byung Koh, Jin Hyeog Lee, Hui‑Yun Jung, Joohyung Ha, Hyung Woo Kim, Jung Tak Park, Seung Hyeok Han, Shin‑Wook Kang, Tae‑Hyun Yoo

**Affiliations:** 1 Department of Internal Medicine, Institute of Kidney Disease Research, Yonsei University College of Medicine, Seoul, Republic of Korea; 2 Department of Internal Medicine, International Saint Mary’s Hospital, Catholic Kwandong University, Incheon, Republic of Korea

Correction to: *Scientific Reports* 10.1038/s41598-024-64224-z, published online 21 June 2024

The original version of this Article contained an error in the Funding section.

“This work was supported by Fresenius Medical Care, Korea (ㅊ).”

now reads:

“This work was supported by Fresenius Medical Care, Korea (2014-KR-008).”

The original Article has been corrected.

