# Supplementary material for: Volume control strategy and patient survival in sepsis-associated acute kidney injury receiving continuous renal replacement therapy: a randomized controlled trial with secondary analysis
Source: Sci Rep. 2024 Jun 21;14:14284. doi: 10.1038/s41598-024-64224-z (PMC11190228; doi:10.1038/s41598-024-64224-z)

**Volume Control Strategy and Patient Survival in Sepsis-associated Acute Kidney Injury Receiving Continuous Renal Replacement Therapy: A Randomized Controlled Trial with Secondary Analysis**

Cheol Ho Park, MD, PhD,^1^ Hee Byung Koh, MD,^2^ Jin Hyeog Lee, MD,^2^ Hui Yun Jung, MD,^1^ Joohyung Ha, MD,^1^ Hyung Woo Kim, MD,^1^ Jung Tak Park, MD, PhD,^1^ Seung Hyeok Han, MD, PhD,^1^ Shin-Wook Kang, MD, PhD,^1^ and Tae-Hyun Yoo, MD, PhD,^1,*^

^1^Department of Internal Medicine, Institute of Kidney Disease Research, Yonsei University College of Medicine, Seoul, Republic of Korea

^2^Department of Internal Medicine, International Saint Mary’s Hospital, Catholic Kwandong University, Incheon, Republic of Korea

**^*^Corresponding author:**

Tae-Hyun Yoo, MD, PhD

Department of Internal Medicine, Institute of Kidney Disease Research, Yonsei University College of Medicine, 50-1 Yonsei-ro, Seodaemun-gu, Seoul, 03722, Republic of Korea.

E-mail: yoosy0316@yuhs.ac

Tel: 82-2-2228-1975

Fax: 82-2-393-6884

**SUPPLEMENTARY MATERIAL**

**Supplementary Table S1.** Comparison of Vasoactive-inotropic Score Status and Vasopressor Dependency Index Status according to Volume Control Strategies

**Supplementary Table S2.** Baseline Characteristics the Study Participants according to the Achieved Volume Accumulation Rate Categories

**Supplementary Table S3.** Patient Survival according to the Achieved Volume Accumulation

**Supplementary Table S4.** Hazard Ratios for Patient Survival Based on Achieved Volume Accumulation

**Supplementary Figure S1.** Kaplan-Meier Curves Showing Patient 7-Day Survival according to Volume Control Strategies

**Supplementary Figure S2.** Kaplan-Meier Curves Showing Patient Survival according to Achieved Volume Accumulation Rate Categories

**Supplementary Figure S3.** Bioelectrical Impedance Analysis-guided Volume Control Strategy

**Supplementary Table S1. Comparison of Vasoactive-inotropic Score Status and Vasopressor Dependency Index Status according to Volume Control Strategies**

|  | Conventional volume control | BIA-guided volume control | *P* value |  |
| --- | --- | --- | --- | --- |
|  |  |  |  |  |
|  | N=39 | N=34 |  |  |
| Vasoactive-inotropic score |  |  |  |  |
| Day 1 | 15.0 (5.2-45.7) | 24.0 (11.4-60.4) | 0.19 |  |
| Day 2 | 6.9 (1.5-30.5) | 10.5 (1.9-28.9) | 0.62 |  |
| Day 3 | 3.9 (0.9-23.7) | 9.5 (3.3-19.3) | 0.21 |  |
| ≥30% increase in vasoactive-inotropic score, n(%) |  |  |  |  |
| Day 1 | 9 (23.1) | 7 (20.6) | 0.80 |  |
| Day 2 | 10 (25.6) | 8 (23.5) | 0.83 |  |
| Day 3 | 11 (28.2) | 11 (32.4) | 0.70 |  |
| ≥50% increase in vasoactive-inotropic score, n(%) |  |  |  |  |
| Day 1 | 8 (20.5) | 5 (14.7) | 0.52 |  |
| Day 2 | 9 (23.1) | 7 (20.6) | 0.80 |  |
| Day 3 | 11 (28.2) | 11 (32.4) | 0.70 |  |
| Vasopressor dependency index, mmHg^-1^ |  |  |  |  |
| Day 1 | 0.2 (0.1-0.6) | 0.3 (0.1-0.8) | 0.23 |  |
| Day 2 | 0.1 (0.0-0.4) | 0.1 (0.0-0.3) | 0.63 |  |
| Day 3 | 0.1 (0.0-0.3) | 0.1 (0.0-0.2) | 0.23 |  |

Data are expressed as mean (standard deviation), median [interquartile range], or count (%).

Abbreviations: BIA, bioelectrical impedance analysis.

**Supplementary Table S2.** **Baseline Characteristics the Study Participants according to the Achieved Volume Accumulation Rate Categories**

|  | Total N=55 | Achieved volume change rate | | | |
| --- | --- | --- | --- | --- | --- |
|  |  | ≤-50% | -50–0% | 1–50% | >50% |
|  |  | N=12 | N=14 | N=13 | N=16 |
| Age, yr | 67 [58–74] | 67 [58–72] | 68 [63–77] | 64 [54–75] | 69 [54–75] |
| Male, n(%) | 32 (58.2) | 5 (41.7) | 10 (71.4) | 9 (69.2) | 8 (50.0) |
| BMI, kg/m^2^ | 23.8 (3.7) | 25.2 (4.5) | 24.3 (4.1) | 23.3 (2.4) | 22.7 (3.7) |
| Comorbid diseases, n(%) |  |  |  |  |  |
| Hypertension | 21 (38.2) | 3 (25.0) | 5 (35.7) | 6 (46.2) | 7 (43.8) |
| Diabetes mellitus | 19 (34.5) | 6 (50.0) | 6 (42.9) | 5 (38.5) | 2 (12.5) |
| Ischemic heart disease | 7 (12.7) | 1 (8.3) | 2 (14.3) | 2 (15.4) | 2 (12.5) |
| Chronic kidney disease | 30 (54.5) | 5 (41.7) | 11 (78.6) | 6 (46.2) | 8 (50.0) |
| Type of infection |  |  |  |  |  |
| Respiratory | 28 (50.9) | 6 (50.0) | 7 (50.0) | 6 (46.2) | 9 (56.3) |
| Gastrointestinal | 12 (21.8) | 1 (8.3) | 3 (21.4) | 5 (38.5) | 3 (18.8) |
| Genitourinary | 3 (5.5) | 2 (16.7) | 0 (0.0) | 1 (7.7) | 0 (0.0) |
| Musculoskeletal | 1 (1.8) | 1 (8.3) | 0 (0.0) | 0 (0.0) | 0 (0.0) |
| Others | 11 (20.0) | 2 (16.7) | 4 (28.6) | 1 (7.7) | 4 (25.0) |
| Mechanical ventilation, n(%) | 47 (85.5) | 11 (91.7) | 11 (78.6) | 11 (84.6) | 14 (87.5) |
| Vasoactive-inotropic score | 24.5 [13.4–63.2] | 20.9 [10.8–71.7] | 14.1 [9.8–20.5] | 21.1 [15.2–48.4] | 57.5 [38.2–86.3] |
| Vasopressor dependency index, mmHg^-1^ | 0.4 [0.2–0.8] | 0.3 [0.1–0.8] | 0.2 [0.1–0.3] | 0.3 [0.2–0.7] | 0.8 [0.5–1.2] |
| APACHE II score | 32 [27–35] | 31 [25–34] | 31 [26–33] | 36 [31–37] | 32 [27–35] |
| SOFA score | 12 [11–15] | 11 [10–15] | 12 [10–14] | 13 [12–15] | 12 [11–15] |
| eGFR, mL/min/1.73m^2^ |  |  |  |  |  |
| Preadmission | 79 [40–99] | 61 [29–92] | 60 [32–81] | 85 [64–112] | 81 [39–104] |
| At CRRT initiation | 23 [14–38] | 19 [14–27] | 24 [12–38] | 26 [20–38] | 29 [13–42] |
| Mean arterial pressure, mmHg | 76 (13) | 77 (11) | 80 (17) | 71 (5) | 74 (14) |
| Urine output for 2 h before CRRT initiation, mL | 20 [5–65] | 28 [3–75] | 21 [10–65] | 20 [0–115] | 10 [3–38] |
| White blood cells, ×1000 cells/μL | 15.89 (11.52) | 16.98 (12.98) | 16.70 (9.08) | 15.78 (9.95) | 14.47 (14.14) |
| Hemoglobin, g/dL | 9.3 (2.0) | 9.5 (1.9) | 9.2 (1.9) | 8.7 (1.7) | 9.6 (2.5) |
| Blood urea nitrogen, mg/dL | 63.3 (31.6) | 70.9 (37.0) | 73.6 (40.2) | 51.2 (20.5) | 58.6 (23.5) |
| Albumin, g/dL | 2.6 (0.4) | 2.6 (0.5) | 2.7 (0.3) | 2.5 (0.4) | 2.5 (0.4) |
| C-reactive protein, mg/dL | 114.8 (110.5) | 53.4 (79.4) | 126.6 (90.9) | 134.1 (110.0) | 140.9 (137.0) |
| pH | 7.28 (0.13) | 7.28 (0.18) | 7.29 (0.15) | 7.30 (0.06) | 7.27 (0.13) |
| Lactate, mmol/L | 4.8 (4.5) | 3.9 (3.0) | 3.1 (3.9) | 6.6 (6.1) | 5.6 (4.2) |
| Volume overload, L | 3.7 [2.4–6.9] | 1.8 [-0.3–3.3] | 7.1 [3.2–7.9] | 4.9 [3.7–7.1] | 3.4 [2.2–5.1] |
| CRRT duration, days | 6.0 [4.0–12.0] | 5.5 [4.0–9.0] | 7.0 [4.0–12.0] | 8.0 [5.0–18.0] | 5.5 [4.0–7.5] |
| BIA-guided volume control, n(%) | 23 (41.8) | 7 (58.3) | 6 (42.9) | 5 (38.5) | 5 (31.3) |
| CRRT ultrafiltration volume, mL/day | 37.5 (3.6) | 36.3 (2.1) | 37.6 (2.9) | 36.5 (4.6) | 39.1 (3.7) |
| CRRT ultrafiltration rate, mL/kg/day | 2473 (1051) | 2914 (1478) | 2643 (779) | 2458 (939) | 2007 (854) |
| CRRT dose, mL/kg/h | 40.3 (17.5) | 46.9 (25.8) | 40.8 (13.9) | 40.3 (16.0) | 34.9 (13.5) |

Data are expressed as mean (standard deviation), median [interquartile range], or count (%).

Abbreviations: APACHE, Acute Physiology and Chronic Health Evaluation; BIA, bioelectrical impedance analysis; BMI, body mass index; CRRT, continuous renal replacement therapy; eGFR, estimated glomerular filtration rate; SOFA, Sequential Oran Failure Assessment.

**Supplementary Table S3. Patient Survival according to the Achieved Volume Accumulation**

| Outcome | Overall | Achieved volume accumulation rate category | | | | *P*-value^*^ |
| --- | --- | --- | --- | --- | --- | --- |
|  |  | ≤-50% | -50–0% | 1–50% | >50% |  |
| Number of subjects | 55 | 12 | 14 | 13 | 16 |  |
| 28-day mortality |  |  |  |  |  |  |
| Person-day | 1087 | 285 | 293 | 320 | 189 |  |
| Events | 26 | 4 | 6 | 3 | 13 |  |
| Events per 1000 person-day | 23.9 | 14.0 | 20.5 | 9.4 | 68.8 | <0.001 |
| 90-day mortality |  |  |  |  |  |  |
| Person-day | 2327 | 693 | 622 | 703 | 309 |  |
| Events | 35 | 5 | 8 | 7 | 15 |  |
| Events per 1000 person-day | 15.0 | 7.2 | 12.9 | 10.0 | 48.5 | <0.001 |
| ICU death |  |  |  |  |  |  |
| Person-day | 1247 | 341 | 468 | 304 | 134 |  |
| Events | 27 | 3 | 7 | 5 | 12 |  |
| Events per 1000 person-day | 21.7 | 8.8 | 15.0 | 16.4 | 89.6 | <0.001 |
| In-hospital death |  |  |  |  |  |  |
| Person-day | 3205 | 810 | 1401 | 683 | 331 |  |
| Events | 39 | 5 | 11 | 8 | 15 |  |
| Events per 1000 person-day | 12.2 | 6.2 | 7.9 | 11.7 | 45.3 | <0.001 |

^*^*P*-value based on log-rank test

Abbreviation: ICU, intensive care unit.

**Supplementary Table S4. Hazard Ratios for Patient Survival Based on Achieved Volume Accumulation**

|  | Achieved volume accumulation rate | | | | | | | | Achieved volume accumulation rate  per 10% increase | |
| --- | --- | --- | --- | --- | --- | --- | --- | --- | --- | --- |
|  | ≤-50% | | -50–0% | | 1–50% | | >50% | |  |  |
|  | HR (95% CI) | *P*-value | HR (95% CI) | *P*-value | HR (95% CI) | *P*-value | HR (95% CI) | *P*-value | HR (95% CI) | *P*-value |
| 28-day mortality | | | | | | | | | | |
| Model 1 | 1.00 | - | 1.42 (0.40–5.03) | 0.589 | 0.67 (0.15–2.99) | 0.600 | 4.96 (1.60–15.38) | 0.005 | 1.07 (1.03–1.11) | 0.001 |
| Model 2 | 1.00 | - | 1.33 (0.34–5.16) | 0.678 | 0.72 (0.15–3.45) | 0.685 | 4.72 (1.28–17.44) | 0.020 | 1.07 (1.02–1.12) | 0.004 |
| Model 3 | 1.00 | - | 0.95 (0.16–5.64) | 0.951 | 0.16 (0.02–1.22) | 0.077 | 6.54 (1.24–34.35) | 0.027 | 1.06 (1.01–1.12) | 0.019 |
| Model 4 | 1.00 | - | 0.90 (0.16–5.02) | 0.901 | 0.21 (0.03–1.61) | 0.132 | 8.08 (1.18–55.50) | 0.034 | 1.06 (0.99–1.12) | 0.076 |
| 90-day mortality | | | | | | | | | | |
| Model 1 | 1.00 | - | 1.65 (0.54–5.04) | 0.383 | 1.33 (0.42–4.20) | 0.624 | 5.62 (2.01–15.66) | 0.001 | 1.07 (1.03–1.11) | <0.001 |
| Model 2 | 1.00 | - | 1.32 (0.40–4.35) | 0.652 | 1.21 (0.36–4.10) | 0.758 | 4.46 (1.37–14.56) | 0.013 | 1.06 (1.02–1.11) | 0.004 |
| Model 3 | 1.00 | - | 1.04 (0.23–4.63) | 0.963 | 0.46 (0.10–2.02) | 0.302 | 5.02 (1.24–20.28) | 0.024 | 1.05 (1.01–1.11) | 0.029 |
| Model 4 | 1.00 | - | 1.21 (0.29–5.01) | 0.797 | 0.55 (0.12–2.48) | 0.435 | 7.18 (1.58–32.51) | 0.011 | 1.05 (1.00–1.11) | 0.048 |
| ICU death | | | | | | | | | | |
| Model 1 | 1.00 | - | 1.96 (0.50–7.67) | 0.334 | 1.43 (0.34–6.00) | 0.629 | 9.93 (2.56–38.48) | 0.001 | 1.09 (1.04–1.14) | <0.001 |
| Model 2 | 1.00 | - | 1.83 (0.45–7.50) | 0.403 | 1.74 (0.38–7.94) | 0.474 | 10.25 (2.38–44.19) | 0.002 | 1.10 (1.04–1.15) | <0.001 |
| Model 3 | 1.00 | - | 1.80 (0.33–9.71) | 0.495 | 0.63 (0.11–3.63) | 0.606 | 15.46 (2.84–84.19) | 0.002 | 1.09 (1.03–1.15) | 0.003 |
| Model 4 | 1.00 | - | 1.69 (0.32–8.86) | 0.534 | 0.66 (0.11–4.04) | 0.656 | 19.33 (2.99–125.08) | 0.002 | 1.09 (1.02–1.16) | 0.012 |
| In-hospital death | | | | | | | | | | |
| Model 1 | 1.00 | - | 1.67 (0.55–5.05) | 0.362 | 1.69 (0.55–5.20) | 0.358 | 6.40 (2.27–18.03) | <0.001 | 1.07 (1.04–1.11) | <0.001 |
| Model 2 | 1.00 | - | 1.45 (0.44–4.73) | 0.541 | 1.71 (0.52–5.65) | 0.381 | 5.71 (1.76–18.53) | 0.004 | 1.07 (1.03–1.11) | 0.001 |
| Model 3 | 1.00 | - | 1.22 (0.29–5.15) | 0.791 | 0.77 (0.19–3.24) | 0.727 | 6.38 (1.62–25.11) | 0.008 | 1.06 (1.02–1.11) | 0.009 |
| Model 4 | 1.00 | - | 1.44 (0.36–5.87) | 0.607 | 0.93 (0.21–4.16) | 0.929 | 8.99 (2.05–39.45) | 0.004 | 1.06 (1.01–1.11) | 0.013 |

Model 1: unadjusted.

Model 2: adjusted for age, sex, BMI, type of infection, and intervention arm.

Model 3: Model 2 + vasopressor dependency index, APACHE II score, fluid overload, and serum lactate level.

Model 4: Model 3 + CRRT ultrafiltration rate and CRRT dose.

Abbreviations: BIA, bioelectrical impedance analysis; CI, confidence interval; CRRT, continuous renal replacement therapy; HR, hazard ratio; ICU, intensive care unit.

**Supplementary Figure S1. Kaplan-Meier Curves Showing Patient 7-Day Survival according to Volume Control Strategies**


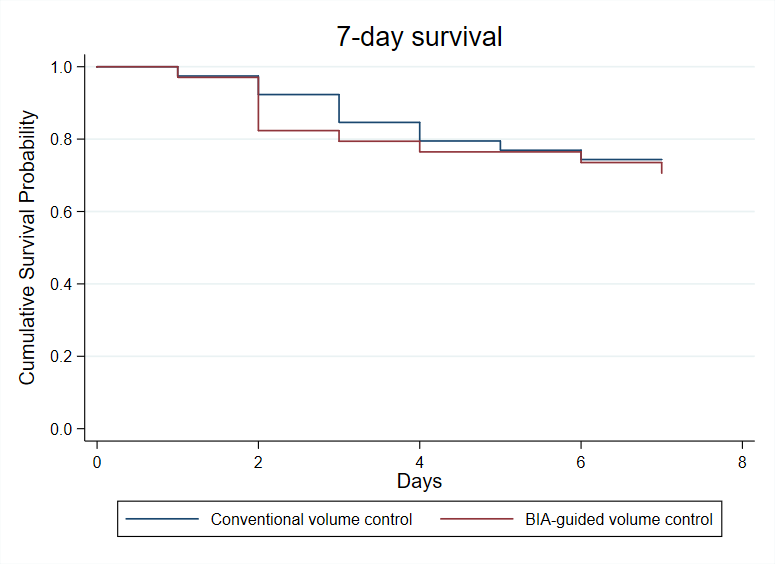


Cumulative survival probability within 7-day of continuous renal replacement therapy initiation according to volume control strategies.

Log-rank tests were used for comparison between groups.

Abbreviations: BIA, bioelectrical impedance analysis.

**Supplementary Figure S2. Kaplan-Meier Curves Showing Patient Survival according to Achieved Volume Accumulation Rate Categories**

**
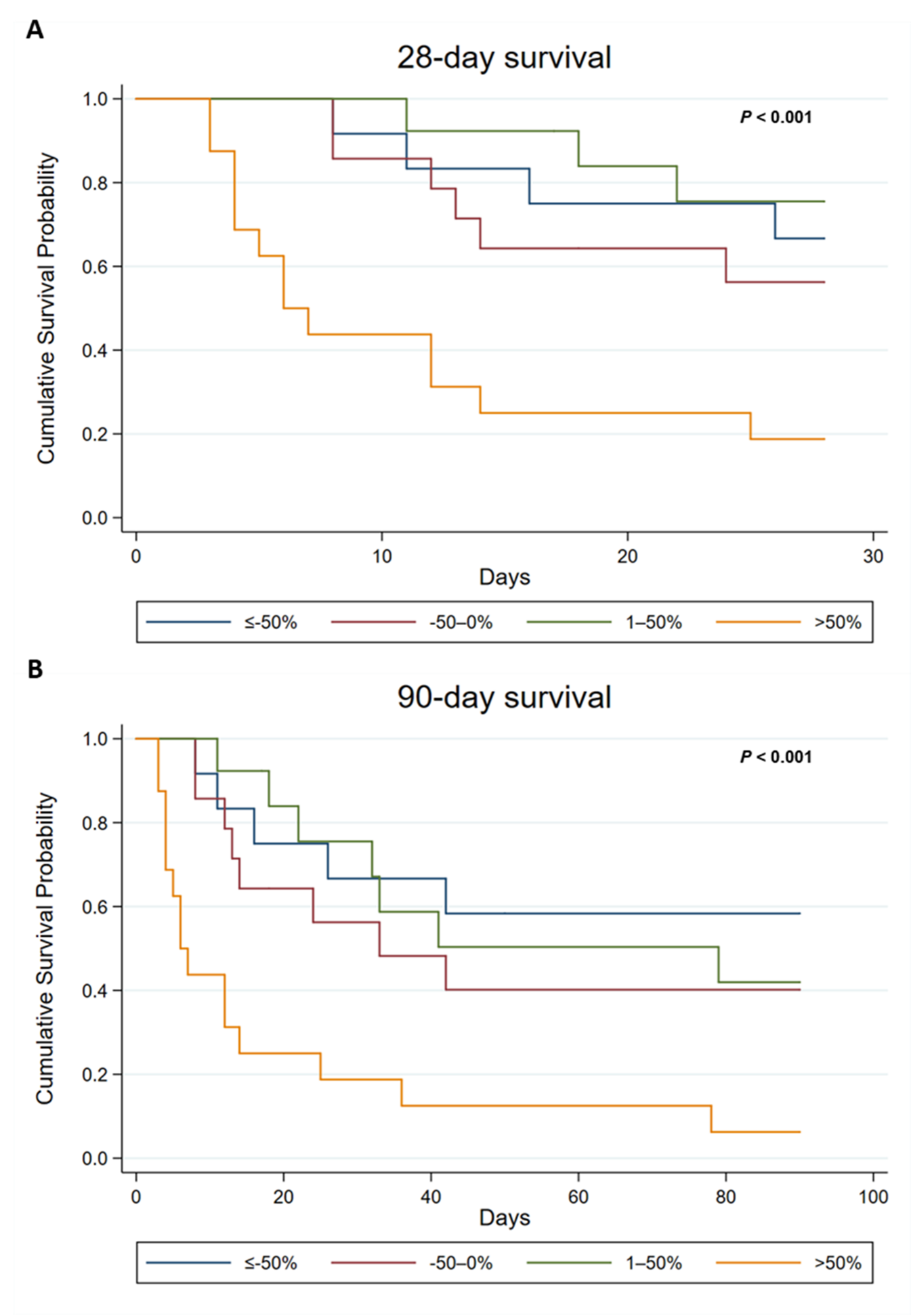
**

Cumulative survival probability within (A) 28-day and (B) 90-day of continuous renal replacement therapy initiation according to achieved volume accumulation rate.

Log-rank tests were used for comparison between groups.

**Supplementary Figure S3. Bioelectrical Impedance Analysis-guided Volume Control Strategy**


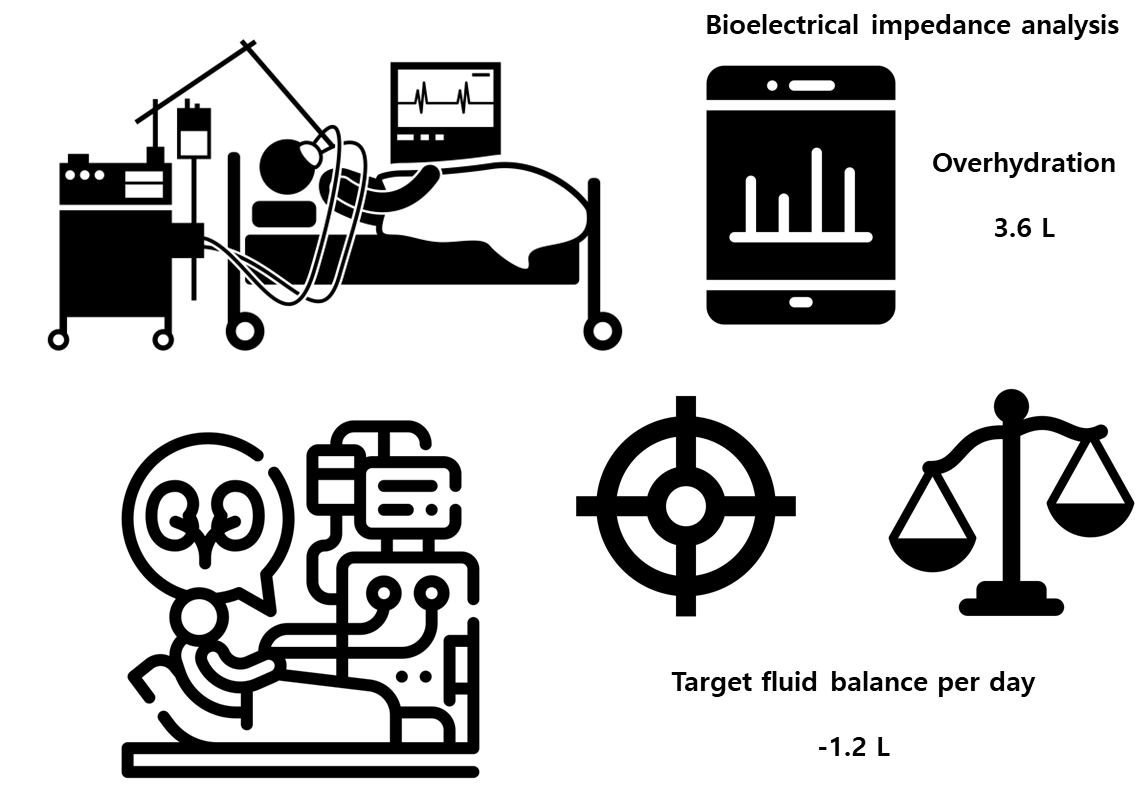

Supplement: Supplementary file 1 — Supplementary Information. [file 41598_2024_64224_MOESM1_ESM.docx]
